# Supplementary material for: Sustained Oropouche virus transmission in Rio de Janeiro’s Atlantic Forest: genomic evidence over a two-year period
Source: Mem Inst Oswaldo Cruz. 2026 Mar 30;121:e250181. doi: 10.1590/0074-02760250181 (PMC13035214; doi:10.1590/0074-02760250181)
Supplement: Supplementary material [file 1678-8060-mioc-121-e250181-s1.pdf]

TABLE I
Novel complete Oropouche virus genomes sequenced from Rio de Janeiro State

ID	Accession ID	Ct	Municipality	Sampling date	Origin
4816_2024	EPI_ISL_19880158	20.89	Piraí	2024-03-24	LACEN/RJ
4827_2024	EPI_ISL_19880159	17.97	Piraí	2024-03-24	LACEN/RJ
5216_2024	EPI_ISL_19880160	25.54	Japeri	2024-03-29	LACEN/RJ
5323_2024	EPI_ISL_19880161	21.12	Piraí	2024-04-02	LACEN/RJ
5844_2024	EPI_ISL_19880163	19.19	Seropédica	2024-04-03	LACEN/RJ
5396_2024	EPI_ISL_19880162	20.76	Piraí	2024-04-06	LACEN/RJ
6305_2024	EPI_ISL_19880164	24.2	Guapimirim	2024-04-15	LACEN/RJ
6408_2024	EPI_ISL_19880165	28.36/28.5	Piraí	2024-04-18	LACEN/RJ
6412_2024	EPI_ISL_19880166	29.63/29.57	Piraí	2024-04-18	LACEN/RJ
6413_2024	EPI_ISL_19880167	27.6/27.47	Piraí	2024-04-18	LACEN/RJ
6676_2024	EPI_ISL_19880168	20.06	Piraí	2024-04-20	LACEN/RJ
8161_2024	EPI_ISL_19880156	25.82/25.97	Bom Jesus do Itabapoana	2024-05-24	LACEN/RJ
8162_2024	EPI_ISL_19880157	23.32/23.36	Mesquita	2024-05-24	LACEN/RJ
8158_2024	EPI_ISL_19880153	28.21/28.56	Paracambi	2024-05-28	LACEN/RJ
8159_2024	EPI_ISL_19880154	29.23/29.46	Angra dos Reis	2024-06-06	LACEN/RJ
8160_2024	EPI_ISL_19880155	24.48/24.54	Guapimirim	2024-06-17	LACEN/RJ
0849_2025	EPI_ISL_19880020	21.86/20.68	Macaé	2025-01-21	LACEN/RJ
0854_2025	EPI_ISL_19880139	21.69/21.76	Petrópolis	2025-01-23	LACEN/RJ
0855_2025	EPI_ISL_19880140	23.66/23.68	Iguaba Grande	2025-01-24	LACEN/RJ
0856_2025	EPI_ISL_19880141	20.51/20.65	Petrópolis	2025-01-24	LACEN/RJ
0851_2025	EPI_ISL_19880136	20.16/20.19	Porciúncula	2025-01-27	LACEN/RJ
0853_2025	EPI_ISL_19880138	21.19/21.21	Cachoeiras de Macacu	2025-01-28	LACEN/RJ
0852_2025	EPI_ISL_19880137	23.49/23.38	Cantagalo	2025-01-28	LACEN/RJ
0857_2025	EPI_ISL_19880142	22.16/22.49	Casimiro de Abreu	2025-01-29	LACEN/RJ
0858_2025	EPI_ISL_19880143	21.88/21.99	Guapimirim	2025-01-29	LACEN/RJ
0850_2025	EPI_ISL_19880135	20.09/19.19	Cachoeiras de Macacu	2025-01-30	LACEN/RJ
0883_2025	EPI_ISL_19880144	21.13/21.05	Japeri	2025-02-10	INI
0886_2025	EPI_ISL_19880145	16.91/16.83	Japeri	2025-02-10	INI
1209_2025	EPI_ISL_19880147	21.76/22.03	Cachoeiras de Macacu	2025-02-14	INI
1217_2025	EPI_ISL_19880148	20.9/20.95	Cachoeiras de Macacu	2025-02-14	INI
1221_2025	EPI_ISL_19880149	19.39/19.62	Cachoeiras de Macacu	2025-02-14	INI
1226_2025	EPI_ISL_19880150	19.31/19.18	Cachoeiras de Macacu	2025-02-14	INI
1201_2025	EPI_ISL_19880151	21.95/22.25	Japeri	2025-02-14	INI
1241_2025	EPI_ISL_19880151	30.14/30.2	Cachoeiras de Macacu	2025-02-17	INI
1273_2025	EPI_ISL_19880152	27.62/27.73	Cachoeiras de Macacu	2025-02-18	INI

Ct: cycle threshold; LACEN/RJ: Central Laboratory of Rio de Janeiro; INI: National Institute of Infectious Diseases.

TABLE II

Genomic coverage of novel Oropouche virus genomes sequenced from Rio de Janeiro State

Accession ID	Collection date	Rio de Janeiro municipality	Small (n)	Small (%)	Medium (n)	Medium (%)	Large (n)	Large (%)
EPI_ISL_19880135	2025-01-30	Cachoeiras de Macacu	671	70	4306	98	6702	98
EPI_ISL_19880136	2025-01-27	Porciuncula	671	70	4306	98	6702	98
EPI_ISL_19880137	2025-01-28	Cantagalo	671	70	4306	98	6702	98
EPI_ISL_19880138	2025-01-28	Cachoeiras de Macacu	417	44	3771	86	6405	93
EPI_ISL_19880139	2025-01-23	Petrópolis	671	70	4306	98	6702	98
EPI_ISL_19880140	2025-01-24	Iguaba Grande	635	66	4295	98	6702	98
EPI_ISL_19880141	2025-01-24	Petrópolis	671	70	4306	98	6702	98
EPI_ISL_19880142	2025-01-29	Casemiro de Abreu	633	66	4306	98	6702	98
EPI_ISL_19880143	2025-01-29	Guapimirim	633	66	4306	98	6702	98
EPI_ISL_19880144	2025-02-10	Japeri	671	70	4306	98	6702	98
EPI_ISL_19880145	2025-02-10	Japeri	671	70	4306	98	6702	98
EPI_ISL_19880146	2025-02-14	Japeri	633	66%	3824	87	4619	67
EPI_ISL_19880147	2025-02-14	Cachoeiras de Macacu	633	66	4283	98	6702	98
EPI_ISL_19880148	2025-02-14	Cachoeiras de Macacu	633	66	4060	93	5837	85
EPI_ISL_19880149	2025-02-14	Cachoeiras de Macacu	670	70	4306	98	6702	98
EPI_ISL_19880150	2025-02-14	Cachoeiras de Macacu	633	66	4306	98	6702	98
EPI_ISL_19880151	2025-02-17	Cachoeiras de Macacu	633	66	4061	93	6661	97
EPI_ISL_19880152	2025-02-18	Cachoeiras de Macacu	633	66	4268	97	6680	97
EPI_ISL_19880153	2024-05-28	Paracambi	671	70	4306	98	6702	98
EPI_ISL_19880154	2024-06-06	Angra dos Reis	671	70	4306	98	6702	98
EPI_ISL_19880155	2024-06-17	Guapimirim	671	70	4306	98	6702	98
EPI_ISL_19880156	2024-05-24	Bom Jesus Itabapoana	671	70	4306	98	6702	98
EPI_ISL_19880157	2024-05-24	Mesquita	671	70	4306	98	6702	98
EPI_ISL_19880158	2024-03-24	Piraí	671	70	4306	98	6702	98
EPI_ISL_19880159	2024-03-24	Piraí	671	70	4306	98	6702	98
EPI_ISL_19880160	2024-03-29	Japeri	671	70	4306	98	6702	98
EPI_ISL_19880161	2024-04-02	Piraíi	671	70	4306	98	6702	98
EPI_ISL_19880162	2024-04-06	Piraí	671	70	4306	98	6702	98
EPI_ISL_19880163	2024-04-03	Seropédica	671	70	4306	98	6702	98
EPI_ISL_19880164	2024-04-15	Guapimirim	671	70	4306	98	6702	98
EPI_ISL_19880165	2024-04-18	Piraí	671	70	4306	98	6702	98
EPI_ISL_19880166	2024-04-18	Piraí	671	70	4306	98	6702	98
EPI_ISL_19880167	2024-04-18	Piraí	671	70	4306	98	6702	98
EPI_ISL_19880168	2024-04-20	Piraí	671	70	4306	98	6702	98
EPI_ISL_19880020	2025-01-21	Macaé	671	70	4298	98	6702	98

TABLE III

Oropouche virus (OROV) complete genomes of the $OROV_{BR-2015-2024}$ clade previously published

Database	From	To	Sampling location	DOI
GISAID (https://gisaid.org/)	EPI_ISL_19706611	EPI_ISL_19706627	ES	NA
	EPI_ISL_19723915	EPI_ISL_19723929		
	EPI_ISL_19810547	EPI_ISL_19810568		
GeneBank (https://www.ncbi.nlm.nih.gov/genbank/)	PP153945	PP154172	AM, AC, RO, RR	10.1038/s41591-024-03300
	PQ064571	PQ065491.		
	PQ073181	PQ073186	ES, PE, PR, RJ, SC	10.1016/S1473-3099(24)00687-X
	PQ156583	PQ156627		
	PQ189413	PQ189445		
	PQ295361	PQ295375		
	OL689332	OL689334	GF	10.3201/eid 2710.204760

AC: Acre; AM: Amazonas; BR: Brazil; GF: French Guiana; NA: not available; PE: Pernambuco; PR: Paraná; RO: Rondônia; RR: Roraima; SC: Santa Catarina.

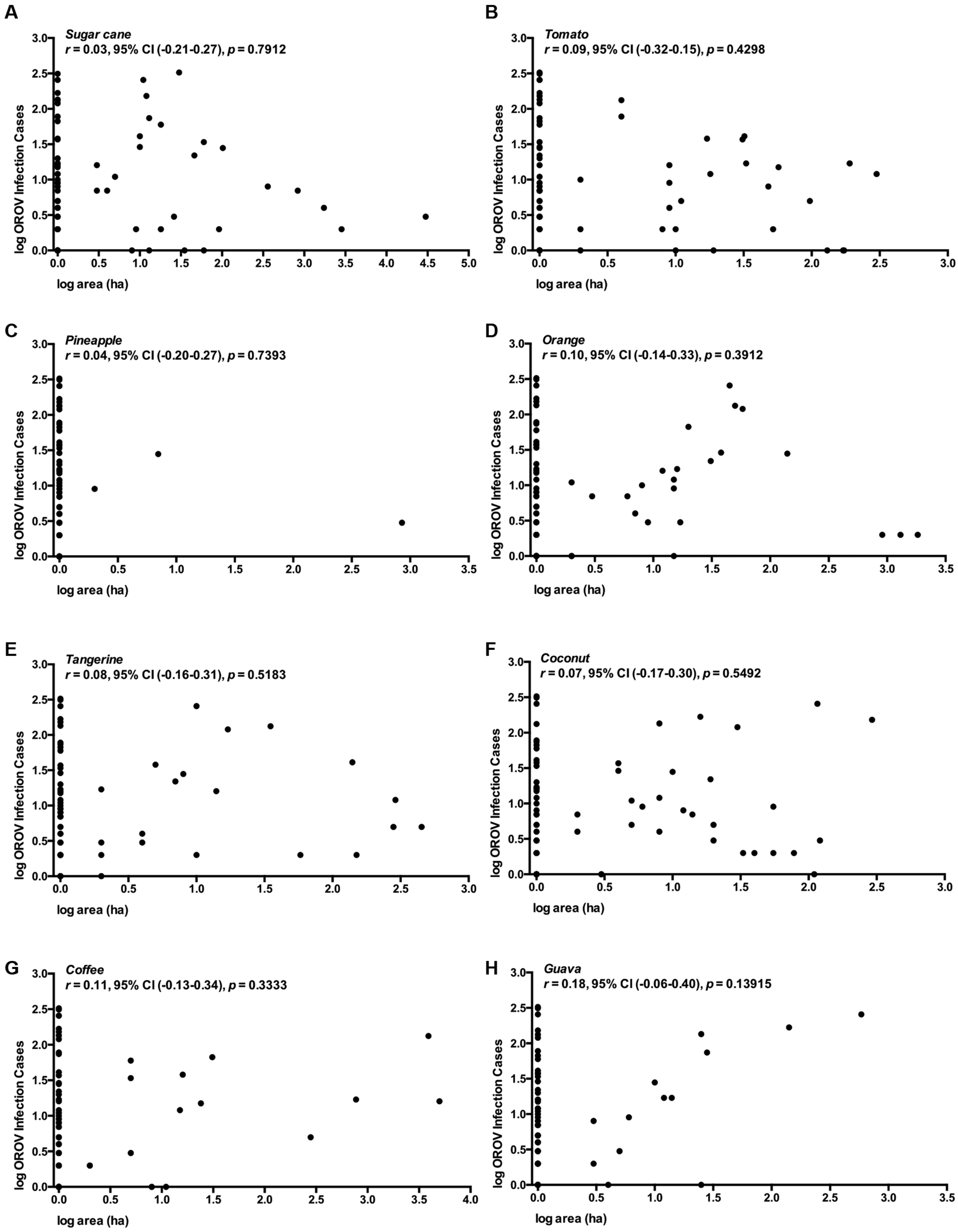

Land use and Oropouche virus (OROV) infection burden in Rio de Janeiro. Spearman correlations between total OROV cases per municipality and total municipal cultivated area dedicated to sugar cane (A), tomato (B), pineapple (C), orange (D), tangerine (E), coconut (F), coffee (G), and guava (H) production. All points represent municipalities; Spearman's ρ and p-values are reported within panels (two-sided).
